# A Fear-Inducing Odor Alters PER2 and c-Fos Expression in Brain Regions Involved in Fear Memory

**DOI:** 10.1371/journal.pone.0020658

**Published:** 2011-05-31

**Authors:** Harry Pantazopoulos, Hamid Dolatshad, Fred C. Davis

**Affiliations:** 1 Department of Biology, Northeastern University, Boston, Massachusetts, United States of America; 2 Laboratory for Translational Neuroscience, Mclean Hospital, Belmont, Massachusetts, United States of America; 3 Department of Psychiatry, Harvard Medical School, Boston, Massachusetts, United States of America; Institut National de la Santé et de la Recherche Médicale, France

## Abstract

Evidence demonstrates that rodents learn to associate a foot shock with time of day, indicating the formation of a fear related time-stamp memory, even in the absence of a functioning SCN. In addition, mice acquire and retain fear memory better during the early day compared to the early night. This type of memory may be regulated by circadian pacemakers outside of the SCN. As a first step in testing the hypothesis that clock genes are involved in the formation of a time-stamp fear memory, we exposed one group of mice to fox feces derived odor (TMT) at ZT 0 and one group at ZT 12 for 4 successive days. A separate group with no exposure to TMT was also included as a control. Animals were sacrificed one day after the last exposure to TMT, and PER2 and c-Fos protein were quantified in the SCN, amygdala, hippocampus, and piriform cortex. Exposure to TMT had a strong effect at ZT 0, decreasing PER2 expression at this time point in most regions except the SCN, and reversing the normal rhythm of PER2 expression in the amygdala and piriform cortex. These changes were accompanied by increased c-Fos expression at ZT0. In contrast, exposure to TMT at ZT 12 abolished the rhythm of PER2 expression in the amygdala. In addition, increased c-Fos expression at ZT 12 was only detected in the central nucleus of the amygdala in the TMT12 group. TMT exposure at either time point did not affect PER2 or c-Fos in the SCN, indicating that under a light-dark cycle, the SCN rhythm is stable in the presence of repeated exposure to a fear-inducing stimulus. Taken together, these results indicate that entrainment to a fear-inducing stimulus leads to changes in PER2 and c-Fos expression that are detected 24 hours following the last exposure to TMT, indicating entrainment of endogenous oscillators in these regions. The observed effects on PER2 expression and c-Fos were stronger during the early day than during the early night, possibly to prepare appropriate systems at ZT 0 to respond to a fear-inducing stimulus.

## Introduction

Circadian rhythms in mammals are regulated by the suprachiasmatic nucleus (SCN) of the hypothalamus, which is optimally positioned above the optic chiasm to receive photic input from the retinohypothalamic tracts [1]. Within individual SCN cells, circadian rhythms are regulated by a positive-negative transcriptional-translational feedback loop consisting of a set of core “clock genes” [2], [3].

Although clock genes regulate circadian rhythms in SCN cells, they are also expressed in cells throughout the brain and body, including the liver, skin, pancreas, lung, heart, and reproductive organs [4], [5], [6], [7], [8], [9], [10], [11], [12], [13], [14], [15]. The functions of clock genes in tissues outside of the SCN has not yet been determined, although some evidence indicates that they are involved in coordinating the daily tasks of the specific tissues in which they are expressed. For example, restricted feeding schedules have been shown to alter the rhythm of clock gene expression in the mouse liver [16], [17] and clock gene expression can directly affect glycogen metabolism in the liver [18]. Bmal1 deficient mice also exhibit altered glucose metabolism [5]. In the lung, evidence indicates that clock gene expression regulates mucin secretion in a circadian manner [10], clock and bmal1 are necessary for regulation of muscle function, and knockout mice for Bmal1 have abnormal electrical and transcriptional retinal responses to light [19].

There is also evidence that circadian rhythms regulate specific behaviors. Honey bees given sugar water at a distinct location during a specific time of day learn to return at the correct time of day to anticipate reward [20]. Food entrainment studies in rodents indicate that the dorsomedial hypothalamic nucleus (DMH) and possibly other brain regions, may be part of a food-entrainable oscillator that functions independently from the SCN, although the role of the DMH in this oscillator remains controversial [21], [22], [23], [24]. Restricted feeding has been shown to alter the rhythm of PER2 protein and induce c-Fos activity during the food availability time in multiple brain regions, including the DMH, the basolateral amygdala (BLA), and the hippocampus (Hipp), but not in the SCN [25], indicating that essential environmental factors such as food availability can alter clock protein expression and cell activity in brain regions other than the SCN. Similar to food entrainment, recent studies have shown that rodents learn to anticipate a foot shock with a specific time of day/night, indicating the formation of a fear-related time-stamp memory [26], [27]. The formation of this memory does not require a functioning SCN [28], indicating the existence of a separate, fear entrained oscillator. In addition, C3H mice entrained at ZT 3 acquired and retained fear memory better than mice entrained at ZT 15, indicating a circadian time effect on fear learning [29]. It is possible that time dependant fear memory may be regulated by circadian pacemakers outside of the SCN.

Although a circadian regulated fear memory and reward memory has been demonstrated in a number of behavioral studies in rodents [26], [27], [29], [30], [31], [32], the cellular mechanisms behind it have not yet been explored. The clock protein period 2 (PER2) is rhythmically expressed in brain regions involved in memory and fear processing, such as the amygdala and the Hipp [33], [34]. Circadian rhythms in neuronal activity have also been reported in a number of brain areas including the Hipp [29] and the piriform cortex (PirCx) [35]. As a first step in testing the hypothesis that, at the cellular level, clock genes in extra-SCN brain regions are involved in the regulation of a time-stamp fear memory, we analyzed the expression of PER2 and c-Fos in the brain regions involved in processing a fear-inducing odor, the SCN, amygdala, Hipp, and PirCx [36], [37], [38], after repeated exposure to a fear-inducing odor at either ZT0 or ZT12.

## Methods

### Animals and Fear Exposure

**Figure 1:**
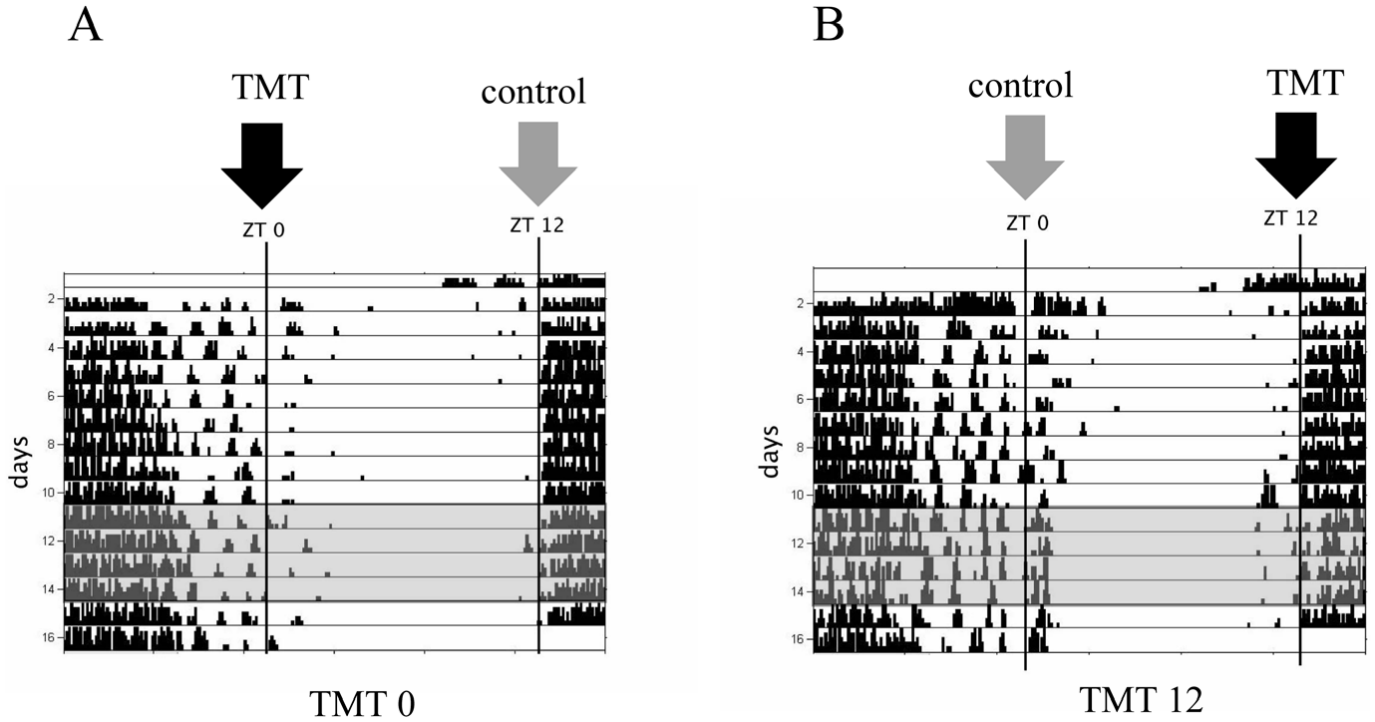
Schedule of TMT exposure. After 10 days of baseline wheel running activity in a LD cycle, one group of mice were exposed to TMT for 4 days at ZT 0 and to a control KimWipe with no odor at ZT 12 (shaded gray area) (A). Animals were then given one full 24 hour cycle without exposure before being sacrificed at 6 hour intervals on the following cycle. A second group of mice were exposed to a control KimWipe at ZT 0 and to TMT at ZT 12 (B).

Adult male C3H mice (36) and male C57BL6 mice (12) were purchased from Charles River Breeding Laboratories (Wilmington, MA). Animals were maintained in a 12∶12 hour light dark (LD) cycle in light-tight black boxes with food and water available ad libitum. ZT 0 was designated as lights on, and ZT 12 was designated as lights off. All experiments were performed according to protocols approved by Northeastern University's Internal Animal Care and Use Committee (protocol # 10-0102 R). One group of C3H (12 mice) was exposed to fox feces derived 2,5-dihydro-2,4,5-trimethylthiazoline (TMT**)** (Contech Inc., Delta, British Columbia) at ZT 0 for 4 successive days (TMT0 group) (Figure 1) by removing the cages from the light tight boxes and transferring them to an adjacent room where a KimWipe with 10 µl of TMT was held above the cage of each animal for 10 minutes. TMT has been established to elicit unconditioned (innate) fear response in rodents [39], [40], [41], [42], [43]. The TMT 0 group was also removed from the light-tight boxes at ZT 12 and brought to the adjacent room where a KimWipe without TMT was held above their cages for 10 minutes as a negative control in order to control for effects of disturbance to the animals at this time point. Lighting conditions in the adjacent room corresponded to the lighting conditions of the animals ZT time, thus animals were never exposed to light outside of the 12∶12 LD cycle. Animals were brought into the adjacent room for exposure to TMT or an empty KimWipe during the light's on portion of the LD cycle, thus the first group, receiving exposure to an empty sterile KimWipe, was brought out 20 minutes before lights off or 20 minutes after lights on, and the second group receiving TMT exposure was brought out 10 minutes before lights off or 10 minutes after lights on. Although animals were disturbed at the time point during which they do not receive TMT, they were not removed from their cages and thus were not introduced to a separate environment. It is important to control for disturbances to animals when analyzing effects related to circadian rhythm, as repeated disturbances to animals may affect circadian rhythm, c-Fos, or clock protein expression regardless of the type of stimulus (fear-inducing vs non fear-inducing). A second group of 12 C3H mice was exposed to TMT at ZT12 (TMT 12) for four successive days and exposed to a negative control KimWipe in the same manner at ZT 0. The group of animals that did not receive TMT at a particular time point was always brought into the adjacent room first, as not to expose the animals to any TMT possibly remaining in the air. The adjacent room was properly ventilated to ensure that TMT was removed from the environment well before the 12 hours after which animals were brought into the room again. This negative control group was used to ensure that any differences observed in Per2 or c-Fos expression would be due to the effect of TMT exposure and not due to general disturbance of the animal. A third group of 12 C3H mice housed in the same conditions but not exposed to TMT and not disturbed by being brought to the adjacent room was included in the study in order to have a group of normal control animals with no disturbances or TMT exposure as an absolute baseline control group.

Animals were euthanized at 4 times of day (ZT 0, ZT 6, ZT 12, ZT 18), in either light or dark conditions depending on ZT time, by cervical dislocation one day after the last exposure to TMT to ensure that any observed changes in PER2 or c-Fos expression would not be related to immediate response to TMT but instead reflect a change in circadian rhythm. One set of 12 male C57BL6 mice (Charles River Breeding Laboratories, Wilmington, MA) were used for a pilot study of quantification of PER2 expression in each brain region. C3H mice were used for the full study to avoid any potential confounding effects that a lack of melatonin in C57BL6 mice may have.

### Tissue Processing and Immunohistochemistry

Whole brains were dissected from mice and post-fixed in 4% paraformaldehyde (PFA) overnight for fixation. Brain samples were then transferred to 20% glycerol in 0.1 M PBS for cryoprotection and were cut into 40 µm serial coronal sections on a freezing microtome (American Optical 860, Buffalo, NY) and stored in 0.1 M PBS with 0.1% NaAzide.

One set of 9 sections from each animal representing the SCN, amygdala, Hipp, and PirCx was chosen for immunostaining for PER2 protein and assigned to 6-well staining dishes. All sections were processed to together for immunohistochemistry to allow for comparison of immunostaining across all sections, brain regions, ZTs, and animals. All solutions were made in Tris buffered saline (pH 7.4) with 0.3% triton-x 100 (TBS-Tx) unless otherwise specified. Sections were washed in TBS-Tx, then incubated in 0.3% H202 for 10 minutes, followed by quick washes in TBS-Tx. Sections were then incubated in blocking solution for 1 hour consisting of 3% normal goat serum (nGs) and 5% milk for PER2. Following several washes in TBS-Tx, sections were placed in primary antibody Rabbit anti-PER2 1∶4000 (Cat# PER21-A; Alpha Diagnostics Inc., San Antonio, TX) with 3% nGs and 5% milk for 72 hours at 4 degrees Celsius. Sections were incubated in biotinylated goat anti-rabbit (1∶200) (Vector Laboratories, Burlingame, CA) in 3% nGs for 2 hours at 4 degrees Celsius followed by washes in TBS and incubation in HRP conjugated streptavidin (Zymed Laboratories, San Francisco, CA) for 2 hours at 4 degrees Celsius. Color reaction was obtained by incubating sections in diaminobenzadine/peroxidase solution (DAB; 0.02%; 0.08% nickel sulfate) in TBS. Sections were then washed in TBS and mounted on gelatin coated glass slides and coverslipped using permount media. Care was taken that each six-well staining dish contained sections from TMT 0, TMT 12, and control samples and was carried through each immunocytochemistry step for the same duration of time to avoid potential sequence effects.

For c-Fos immunohistochemistry, a separate set of 9 sections containing the SCN, amygdala, Hipp, and PirCx were selected and placed into 6-well staining dishes as described for PER2 immunohistochemistry. All solutions were made in 0.1 M PBS with 0.2% triton-x 100 unless otherwise noted. Free-floating sections were washed in PBS-TX, incubated in 0.3% hydrogen peroxide for 10 minutes, followed by quick washes in PBS-Tx. Sections were then incubated in 2% bovine serum albumin (BSA) for 1 hour followed by washes in PBS-Tx, and then incubated in primary antibody rabbit polyclonal anti-c-Fos (1∶30,000) (AB-5; Oncogene Research Products, Cambridge, MA) for 72 hours at 4 degrees Celsius. Sections were then incubated in biotinylated goat anti-rabbit (1∶500) (Vector Laboratories, Burlingame, CA) for 2 hours at room temperature, followed by washes in PBS-TX and incubation in HRP conjugated streptavidin (Zymed Laboratories, San Francisco, CA) for 2 hours at room temperature. Sections were developed in DAB as described for PER2 and mounted on gelatin coated glass slides and coverlsipped using permount media. Care was taken that each six-well staining dish contained sections from TMT 0, TMT 12, and control samples and was carried through each immunocytochemistry step for the same duration of time to avoid potential sequence effects.

### Thionin Nissl Stain

For Nissl staining, 40 µM sections were mounted on gelatin coated glass slides, hydrated through a series of ethanol concentrations, and incubated in thionin stain solution for 10 minutes, dehydrated through a series of ethanol concentrations and coverslipped with permount media.

### Data Analysis

#### Anatomical Divisions

The SCN was outlined based on cytoarchitectonic criteria from Nissl stained sections as well as on PER2 immunostaining using the Chemoarchitectonic Atlas of the Mouse Brain (Paxinos & Watson 2009). Three sections per animal were used for quantification, representing the dorsal, middle, and caudal portions of the SCN. The amygdala was defined by cytoarchitectonic criteria according to the Chemoarchitectonic Atlas of the Mouse Brain (Paxinos & Watson 2009), using PER2 immunostaining as well as Nissl stained sections from the same animals. The amygdala was divided into the lateral, basolateral, and central nuclei. Data for PER2 and c-Fos did not differ between the lateral and basal nuclei, therefore they were grouped together and referred to as the BLA. Data for the central amygdala, due to its separate inputs and function and reports that its rhythm in PER2 expression is anti-phase to the BLA, is reported separately [33], [44], [45], [46]. The medial, capsular, lateral, and lateral capsular divisions of the central amygdala were grouped together for analysis as the central amygdala (CE). Six sections from each animal were chosen for the amygdala, representing the rostral to caudal extent of the region. The amygdala from each hemisphere per section was quantified, resulting in a total of 12 amygdala/animal. The PirCx was identified in the same sections containing the amygdala according to cytoarchitectonic criteria. The portion of the PirCx chosen for quantification consisted of the region that was present in each of the sections chosen for amygdala quantification, and included the deep and superficial layers. This resulted in quantification of 12 PirCx areas/animal. For the Hipp, 6 sections per animal were chosen for quantification, and the Hipp from each hemisphere was quantified, resulting in 12 hippocampi/animal. The layers of the Hipp (stratum oriens-lacunosum moleculare, stratum pyramidale, and stratum radiatum) were difficult to accurately identify on PER2 sections and thus were not subdivided for quantification. The Hipp was divided into the cornu ammonis (CA) regions CA4, CA2/3, and CA1 according to cytoarchitectonic criteria. No differences between subregions were detected therefore the data were combined and reported for the combined Hipp.

#### Quantification of immunostaining

Microscopic images were captured using a Nikon Eclipse E400 microscope with an Olympus DP12 (DIH032861) camera interfaced with Magna-Fire SP 2.1B imaging software. For each region of interest, images were taken at 4x magnification for identification of regions, and 20x magnification for quantification of immunostaining. Immunostaining was quantified using ImageJ 1.41 (N.I.H., U.S.A.). A pilot study was conducted on a set of control sections (9 sections/mouse, 3 mice/timepoint for ZT 0, 4, 12, 18) from male C57BL6 mice to determine the optimal quantification method. For the pilot study, PER2 was quantified using three different methods in ImageJ. The first method was by manually counting all immunoreactive cells within a traced region of interest to obtain a density (number of immunoreactive (IR) cells/area in µm^2^). The second method was by using the thresholding feature in ImageJ after converting all photos to grayscale, to threshold the immunoreactive particles and set shape parameters for the program to determine what to consider a cell. This method automatically counted the number of particles (cells) within a traced region and resulted in IR-cells/area (μm^2^). The third method used the same thresholding technique but instead quantified the total amount of thresholded immunoreactive area. This amount was divided by the total area of the traced region to obtain a density of immunoreactivity (IR-area (μm^2)^/total area (μm^2^)). Threshold settings were held constant across all sections and light intensity was held constant during photo acquisition. All three methods resulted in similar data, with clear rhythms in PER2 expression in the SCN peaking at ZT12 and antiphase rhythms in the BLA, Hipp, and PirCX. The standard error was lowest in the third method, using thresholded IR-area, and was thus chosen as the method reported for the full study. Values for thresholded IR-area for each region were combined across sections, and divided by the total area of the region added across sections to obtain one density value per region per animal for analysis.

#### Wheel running activity

One animal/ time point (ZT 0, 6, 12, 18) for each of the experimental groups (TMT0 & TMT12) was kept in a mouse wheel running cage to monitor activity in order to ensure that mice were trained as expected and that ZTs represented the expected CTs.

### Statistical Analysis

One-way ANOVAs were performed with JMP v5.0.1a (SAS Institute, Cary, North Carolina). For comparisons between groups, for each brain region, the values of thresholded IR-area per section were added across sections from the same animal to obtain one thresholded IR-area value per animal. This value was divided by the sum of the areas of all of the sections for each animal in order to obtain one density value (Thresholded IR-area/total area) for each animal, which was used for group comparisons. For example, for the BLA of one animal: 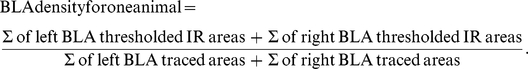



## Results

### Repeated TMT exposure does not affect PER2 or c-Fos expression in the SCN

**Figure 2:**
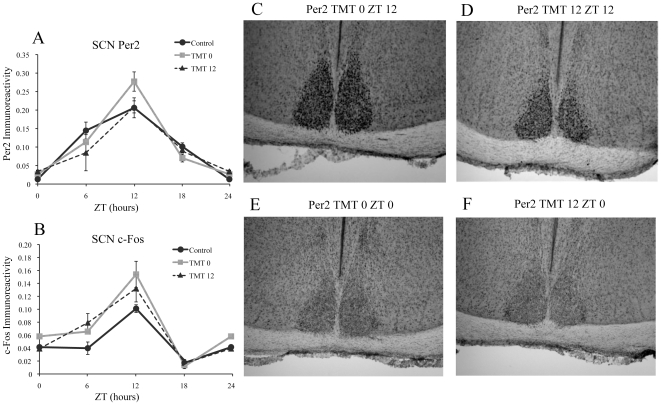
TMT exposure does not affect PER2 or c-Fos expression in the SCN. PER2 expression in the SCN was identical for all three groups (A), with expression peaking at ZT 12. (B) c-Fos immunoreactivity was similar to PER2 in the SCN for all three groups. Photomicrographs showing high PER2 expression in the SCN at ZT 12 for the TMT0 group (C) and the TMT12 group (D), and low PER2 expression at ZT 0 for the TMT0 group (E) and the TMT12 group (F). *significantly different from control group at that time point (p<0.05) **significantly different from control and experimental group at that timepoint (p<0.05). The values for ZT 0 are repeated as ZT 24. Error bars represent standard errors.

Repeated exposure to TMT at either ZT 0 (TMT0) or ZT 12 (TMT12) for four successive days followed by one day without exposure (Figure 1) had no significant effect on the amount PER2 or c-Fos expression when each experimental group was compared to the baseline control group or to each other at each time point (Figure 2). In addition, TMT exposure did not affect the normal rhythm of PER2 or c-Fos expression in the SCN, as a strong effect of ZT was detected in each group (Tables 1 & 2). Identical rhythms in PER2 and c-Fos expression were observed in each group, with a peak of expression for both markers occurring at ZT 12 (Figure 2 A&B).

**Table 1:** One-Way ANOVA (main effect of ZT).

Marker	Condition	Brain region	F-value	p-value
Per2	Control	SCN	82.98	p<0.001
Per2	TMT 0	SCN	28.50	p<0.001
Per2	TMT 12	SCN	52.99	p<0.001
Per2	Control	Basolateral amygdala	52.70	p<0.001
Per2	TMT 0	Basolateral amygdala	10.47	p<0.01
Per2	TMT 12	Basolateral amygdala	1.91	N.S.
Per2	Control	Central Amygdala	40.35	p<0.001
Per2	TMT 0	Central Amygdala	4.26	p<0.05
Per2	TMT 12	Central Amygdala	2.61	N.S.
Per2	Control	Hippocampus	6.47	p<0.01
Per2	TMT 0	Hippocampus	9.01	p<0.01
Per2	TMT 12	Hippocampus	4.86	p<0.05
Per2	Control	Piriform Cortex	4.30	p<0.05
Per2	TMT 0	Piriform Cortex	11.63	p<0.01
Per2	TMT 12	Piriform Cortex	3.97	p<0.05

**Table 2:** One-Way ANOVA (main effect of ZT).

Marker	Condition	Brain region	F-value	p-value
c-Fos	Control	SCN	17.53	p<0.001
c-Fos	TMT 0	SCN	32.07	p<0.001
c-Fos	TMT 12	SCN	15.91	p<0.001
c-Fos	Control	Basolateral amygdala	23.06	p<0.001
c-Fos	TMT 0	Basolateral amygdala	16.31	p<0.001
c-Fos	TMT 12	Basolateral amygdala	21.59	p<0.001
c-Fos	Control	Central Amygdala	16.09	p<0.001
c-Fos	TMT 0	Central Amygdala	10.29	p<0.01
c-Fos	TMT 12	Central Amygdala	8.81	p<0.01
c-Fos	Control	Hippocampus	84.87	p<0.001
c-Fos	TMT 0	Hippocampus	31.15	p<0.01
c-Fos	TMT 12	Hippocampus	45.60	p<0.01
c-Fos	Control	Piriform Cortex	2.15	N.S.
c-Fos	TMT 0	Piriform Cortex	9.39	p<0.01
c-Fos	TMT 12	Piriform Cortex	20.58	p<0.001

### Repeated exposure to TMT alters expression of PER2 and c-Fos in the BLA

**Figure 3:**
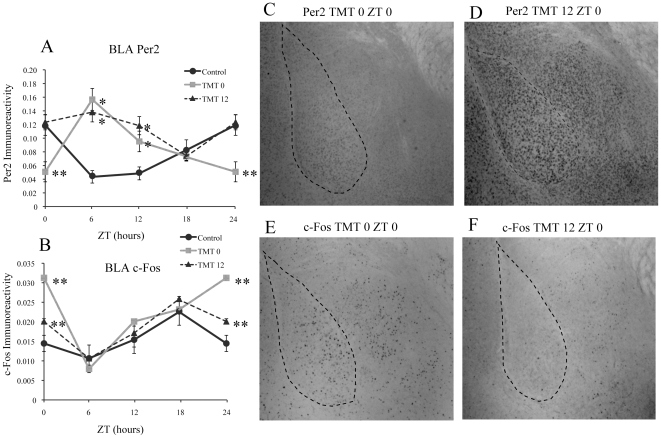
TMT exposure alters expression of PER2 and c-Fos in the BLA. TMT altered the rhythm and amount of expression of PER2 in the BLA resulting in significantly lower expression at ZT 0 for the TMT0 group and significantly higher expression at ZT 6 and ZT 12 for both experimental groups (A). TMT exposure also resulted in an increase of c-Fos expression at ZT 0 for both groups, with the increase for the TMT0 group significantly higher than the TMT12 group (B). Photomicrographs showing low amount of PER2 immunoreactivity in the BLA of a TMT 0 animal at ZT 0 (C) and normal amount of immunoreactivity the same timepoint for a TMT12 animal (D). Photomicrographs showing increased c-Fos expression at ZT 0 in the BLA of a TMT0 animal (E) and lower level of expression at ZT 0 in a TMT12 animal (F). *significantly different from control group at that time point (p<0.05) **significantly different from control and experimental group at that timepoint (p<0.05). The values for ZT 0 are repeated as ZT 24. Error bars represent standard errors.

Repeated exposure to TMT at ZT 0 essentially reversed the normal rhythm of PER2 expression in the BLA (Figure 3 A, C & D, Table 1) with a peak of expression occurring at ZT 6 and the lowest point of expression at ZT 0. The amount of PER2-IR was significantly lower than control mice at ZT 0, and significantly higher than control mice at ZT 6 and ZT 12 (Figure 3 A). In comparison, results for the TMT12 group were different. PER2 in the TMT12 group remained at control levels in the BLA at ZT 0 and ZT 18 but did not have the normal decrease in PER2 expression at ZT 6 and ZT12, which resulted in essence, a lack of rhythmic PER2 expression, with expression remaining relatively high at all timepoints. (Figure 3, Table 1). The amount of PER2-IR was significantly higher at ZT 0 compared to the TMT0 group, and significantly higher at ZT 6 and ZT 12 compared to the control group (Figure 3 A). Changes in PER2 expression in response to TMT exposure were accompanied by increased c-Fos expression only at ZT 0 for both experimental groups, with the increase in the TMT0 group significantly greater than both the control and TMT12 groups (Figure 3 B). The rhythm of c-Fos expression in the BLA however was not affected by TMT exposure (Table 2).

### Exposure to TMT Alters Rhythm of PER2 and c-Fos Expression in the Central Amygdala

**Figure 4:**
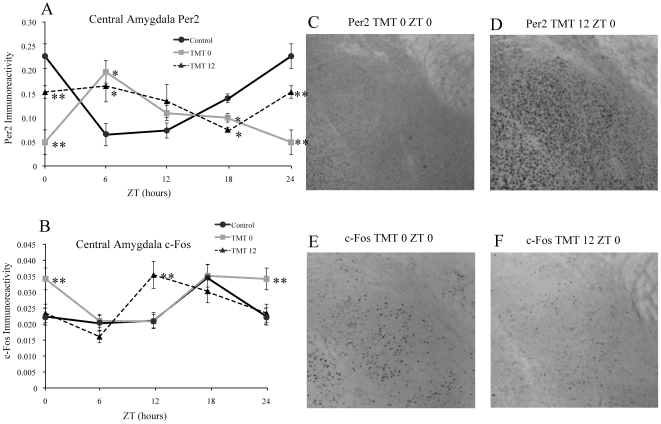
TMT exposure differentially affects PER2 and c-Fos in the central amygdala (CE) based on time of exposure. TMT exposure altered the rhythm and amount of PER2 expression in the CE amygdala of both groups, resulting in a low level of expression at ZT0 in the TMT0 group, followed by a high level of expression at ZT 6 for both groups, and a low level of expression at ZT 18 for both groups (A). TMT resulted in increased c-Fos expression at ZT 0 only for the TMT0 group, and increased expression at ZT 12 only for the TMT12 group (B). Photomicrographs showing decreased PER2 immunoreactivity in the CE of a TMT0 animal at ZT 0 (C) in comparison to a TMT12 animal (D). Photomicrographs depicting increased c-Fos expression at ZT 0 for a TMT0 animal (E) in comparison to a TMT12 animal (F). *significantly different from control group at that time point (p<0.05) **significantly different from control and experimental group at that timepoint (p<0.05). The values for ZT 0 are repeated as ZT 24. Error bars represent standard errors.

Similar to the BLA, exposure to TMT reversed the normal rhythm of PER2 expression in the CE of the TMT0 group, with a peak of PER2 expression occurring at ZT 6 and the low point occurring at ZT 0, with significantly lower PER2-IR than both the control and TMT12 group at ZT 0 (Figure 4 A, C & D, Table 1). Results for the TMT12 group in the CE were similar to results observed in the BLA, with a loss of rhythm in PER2 expression and significantly lower expression at ZT 0 when compared to controls (Figure 4 A, Table 1). Also similar to results in the BLA, c-Fos expression in the TMT0 group was significantly increased only at ZT 0 compared to both control and TMT12 mice, but the rhythm in c-Fos expression was not affected in the TMT0 group (Figure 4, Table 2). In contrast to the effect of c-Fos observed in the BLA, c-Fos expression in the TMT12 group was not increased at ZT 0, it was however significantly increased at ZT 12 compared to both the control and TMT0 groups (Figure 4 B).

### Exposure to TMT Alters PER2 Expression in the Hippocampus

**Figure 5:**
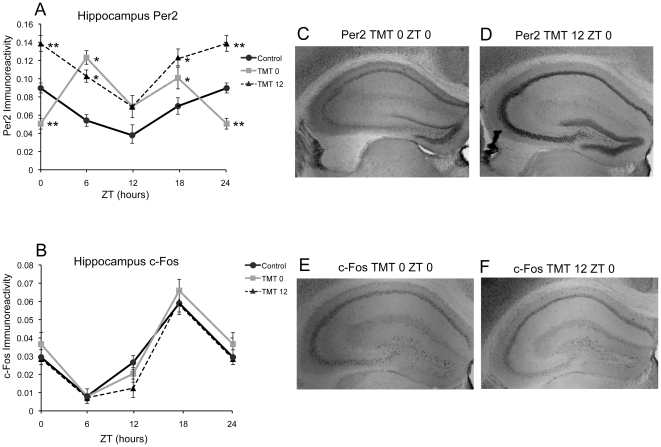
PER2 and c-Fos in the hippocampus is minimally affected by TMT exposure. TMT exposure resulted in overall higher expression of PER2 for both experimental groups, with the exception of lower expression at ZT 0 for the TMT0 group (A). In contrast, c-Fos expression in the Hipp is not affected by TMT exposure (B). Photomicrographs showing less PER2 immunoreactivity at ZT 0 in the Hipp of a TMT0 animal (C) and high amount of immunoreactivity in the Hipp of a TMT12 animal (D). Photomicrographs showing similar amount of c-Fos immunoreactivity in the Hipp of a TMT0 (E) and a TMT12 animal (F). *significantly different from control group at that time point (p<0.05) **significantly different from control and experimental group at that timepoint (p<0.05). The values for ZT 0 are repeated as ZT 24. Error bars represent standard errors.

Similar to the effects of TMT exposure in the BLA and CE, PER2 expression in the Hipp of the TMT0 group was significantly decreased at ZT 0 but significantly increased at ZT 6 compared to control subjects (Figure 5 A, C & D). PER2 expression was still rhythmic in the TMT0 group (Table 1). Unlike the BLA and CE, PER2 expression in the Hipp of the TMT12 group was still rhythmic (Table 1). The amount of PER2 expression however was significantly higher in the TMT12 group compared to the control group at every timepoint except for ZT 12 (Figure 5). The rhythm and amount of c-Fos expression were not affected in the Hipp of either experimental group in comparison to the control mice (Figure 5 B, Table 2).

### TMT Exposure Alters the Rhythm of PER2 Expression and Induces a Rhythm in c-Fos Expression in the Piriform Cortex

**Figure 6:**
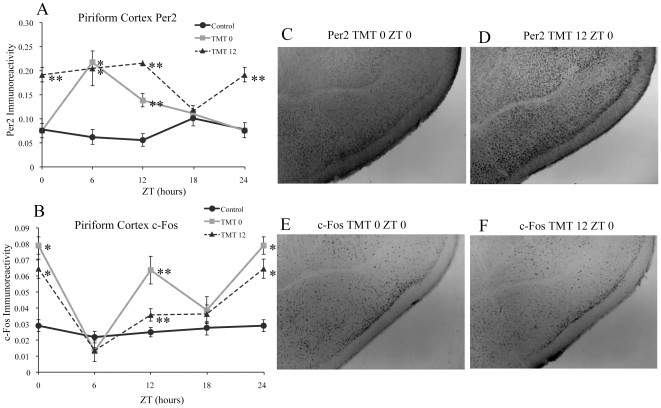
TMT exposure alters PER2 expression and induces a rhythm in c-Fos expression in the Piriform Cortex (PirCx). TMT exposure increased the amount of PER2 expression at ZT 0 for the TMT12 group and at ZT 6 and ZT 12 for both experimental groups in the PirCx (A). Expression of c-Fos was increased at ZT 0 and ZT 12 for both experimental groups, with the increase at ZT 12 for the TMT0 group significantly greater than the TMT12 group (B). Photomicrographs showing normal level of PER2 expression in the PirCx of a TMT0 animal at ZT 0 (C) and higher level of expression at the same timepoint in a TMT12 animal (D). Photomicrographs showing high level of expression of c-Fos at ZT 0 for a TMT0 animal (E) and a TMT12 animal (F). *significantly different from control group at that time point (p<0.05) **significantly different from control and experimental group at that timepoint (p<0.05). The values for ZT 0 are repeated as ZT 24. Error bars represent standard errors.

In contrast to other brain regions, TMT exposure did not decrease PER2 expression at ZT 0 in the TMT0 group. Instead, TMT exposure resulted in significant increase of PER2 expression at ZT 6 and ZT 12 in the TMT0 group compared to control mice (Figure 6 A, C & D). This resulted in rhythmic PER2 expression that is somewhat anti-phase to control animals (Figure 6A, Table 1). In the TMT12 group, TMT exposure resulted in significantly increased PER2 expression at every time point except for ZT 18 when compared to control animals (Figure 6). Although there was no significant rhythm in c-Fos expression in control mice (Figure 6B, Table 2), TMT exposure resulted in an induction of rhythmic c-Fos expression in both experimental groups (Figure 6B, Table 2). The amount of c-Fos expression was significantly increased at ZT0 and ZT12 in both groups in comparison to the control group (Figure 6B, E & F). C-Fos expression at ZT 12 was also significantly increased in the TMT0 group when compared to the TMT12 group (Figure 6B).

### Wheel Running Activity

For recording of wheel-running activity, 4 mice for the TMT0 group and 4 mice for the TMT12 group were housed in running wheel cages during the experiment. All animals were properly entrained to the 12∶12 LD cycle, and TMT exposure in the LD cycle had no obvious effects on wheel-running activity, but the sample size was too small to rule out potential effects of TMT exposure on activity.

## Discussion

Our results show that repeated exposure to a fear-inducing stimulus alters the rhythm of PER2 expression in brain regions responsible for processing this information, but does not affect PER2 expression in the SCN. The lack of effect on the SCN along with the observed effects on PER2 in the other brain regions highlights the role of the SCN as a pacemaker for the animal as a whole, as well as the possibility that other brain regions may be able to act as function-specific pacemakers with some degree of independence from the SCN in a LD cycle in vivo in response to environmental factors. Our results are consistent with data showing no effect of restricted feeding on PER2 or c-Fos expression in the SCN but a strong effect on PER2 and c-Fos in brain regions that may be involved in feeding, memory, and reward processing including the DMH, the Hipp, and the CE and BLA nuclei of the amygdala [25]. Similar to the restricted feeding study, TMT exposure in our study resulted in increased c-Fos expression dependant upon the experimental group and ZT of assessment, indicating that changes in PER2 expression were accompanied by changes in brain activity in response to entrainment to TMT and not as an immediate response to TMT exposure itself, as changes were observed 24 hours after the last exposure to TMT.

The rhythms of Per2 expression in control mice in our study are consistent with previous reports of Per2 rhythms in the SCN, BLA, and Hipp [47], [48], [49]. In the CE, a rhythm of PER2 expression in control mice was not anti-phase to the BLA, as reported in rats and mice [33], [44]. This may be due to a difference between the mouse strains (C3H and C57BL6) that we examined, compared to the rodents used in those studies. Per2 rhythm in the PirCx to our knowledge had not been reported previously.

The rhythm in c-Fos expression in the SCN in our study in control mice is identical to the spontaneous rhythm in C57/BL6 mice reported by Grenados-Fuentes et al. [35] with a peak expression at ZT 12 and a low point of expression between ZT 20-ZT0, and similar to the rhythm reported by Hughes et al. [50], which reported high levels of c-Fos during the day and low levels during the night in animals in a 12∶12 LD cycle. Other studies however report rhythms of c-Fos expression in the SCN that show higher expression at CT 2 and lower expression at CT 14 [51], [52]. Both of these studies however, one in SAMP8 mice [51], and the other in male Wistar rats [52], allowed the animals to free run in DD for a certain period before sacrificing them in darkness for immunohistochemistry. In our study, animals remained on the 12∶12 LD cycle and were sacrificed either in light or dark conditions depending on the ZT time of sacrifice. It is possible that differences in LD conditions, sacrifice conditions, and species and strain may account for differences in c-Fos expression rhythms in the SCN across these studies. The rhythms in c-Fos expression in the BLA, CE, and PirCx in our study in control mice are similar to previously reported rhythms in these regions [23], [25], [35].

### Design Limitations

The negative control condition in our study of disturbing the animals at the opposite time point of TMT exposure with a sterile, empty KimWipe was used to ensure that any differences observed in Per2 or c-Fos expression would be due to the effect of TMT exposure and not due to general disturbance of the animals. Although it is possible that animals may learn to associate a fear for TMT at both time points, work in hamsters has shown that animals learn to associate a fearful stimulus at the circadian time of exposure to the stimulus and not at the opposite timepoint 12 hours later [27], thus it is likely that fear response to repeated TMT exposure is stronger at the ZT time of exposure than at the opposite time when animals where disturbed without exposure to TMT. In either case, our study is designed to test circadian responses to a fearful stimulus at a specific ZT time, thus potentially examining the cellular responses to “time-stamp fear memory” and not necessarily classical fear entrainment. Taking this into consideration however, a third group of 12 C3H mice housed in the same conditions but not exposed to TMT and not disturbed by being brought to the adjacent room was included in the study in order to have a group of normal control animals with no disturbances or TMT exposure as an absolute baseline control group. Since animals were disturbed every 12 hours but exposed to a fear-inducing stimulus only at ZT 0 or ZT 12, we can confidently distinguish effects of the fear-inducing stimulus at a particular ZT time from effects of disturbing the animals. The third group of animals with no disturbance or TMT exposure also provides a negative control to potential effects of disturbance to the animals.

In both experimental groups however, it is important to consider the possibility that the negative control of disturbing the same animals at the opposite time point, 12 hours apart, without exposure to TMT can induce conditioned fear at both the time of exposure and the negative control time of disturbance. This possibility should be taken into consideration when interpreting the data. Future work will examine the effect of repeated exposure to TMT without disturbance of the animals at the opposite timepoint in order to explore this possibility.

### Piriform Cortex

The PirCx receives olfactory input from the olfactory bulbs, and is essential in processing olfactory information [36], [53], [54], [55], [56]. This region was the only area other than the SCN where PER2 expression was not decreased at ZT 0 in comparison to control mice, (Figure 6) although expression was increased at ZT 6 and the overall rhythm was altered in comparison to controls similar to the BLA, CE, and Hipp. In addition, c-Fos expression was increased at both ZT 0 and ZT 12 for both experimental groups, indicating that repeated TMT exposure induced rhythmic expression equally at both timepoints, regardless of the time of TMT exposure. This data corresponds with previous work showing that exposure to cedar wood oil evoked robust rhythmic expression of c-Fos in the PirCx even in SCN lesioned mice [35], but this region does not differentiate between a fearful and non-fearful odor, as cedar wood oil and fox feces equally increased c-Fos in the PirCx. Fox urine however resulted in a greater increase of c-Fos in the BLA compared to cedar wood oil [57].

### Hippocampus

The Hipp is responsible for long term memory formation, storage, and retrieval [58], [59], [60], and has been shown to have rhythmic neuronal firing activity in synchrony with the amygdala during retrieval of fear memory [38], indicating that these two regions work together to process fear memory information. Electrophysiological work has shown that LTP in the Hipp is higher during the subjective night than during the subjective day [61]. In addition, PER2 has been shown to be rhythmically expressed in the Hipp of mice, even in culture, and mice lacking PER2 have abnormal LTP and deficits in memory retrieval [34]. A recent study has shown that phase shifting mice using light pulses disrupts recall of conditioned fear memory [31]. Despite the evidence for circadian regulation of memory formation, we did not see strong effects of TMT exposure on PER2 or c-Fos expression in this region. Although PER2 expression was overall higher in both experimental groups in comparison to control mice, with the exception of a significant decrease at ZT 0 for the TMT0 group, the rhythm of PER2 expression was otherwise unaffected, and c-Fos expression was not affected in any way. Taken together, these results indicate that the Hipp is minimally affected at only ZT 0 by exposure to a fear-inducing odor. Recruitment of the Hipp may not be necessary for processing time-stamp innate fear memory. This type of memory processing may instead occur primarily in the BLA. A number of studies indicate that encoding, reconsolidation, and retrieval of fear memory occurs in the amygdala itself [62], [63], [64], [65], [66].

### Amygdala

The BLA is the brain region responsible for the expression and to some extent the storage, encoding, retrieval, and reconsolidation of fear memory [37], [66], [67]. It receives olfactory information indirectly from the PirCx and entorhinal cortex [68], [69], [70] and sends reciprocal projections to the Hipp for memory processing through a direct pathway and an indirect pathway via the entorhinal cortex [71], [72], [73], [74]. It is also essential for conditioned odor aversion memory consolidation [75]. Repeated TMT exposure lead to a sharp decrease of PER2 expression in the BLA at ZT0 in the TMT0 group, which was accompanied by increased c-Fos expression at the same timepoint, indicating that lower PER2 is associated with increased activity in this region. In control animals, c-Fos expression indicates an increase of activity of this region between ZT12 and ZT18, during the dark cycle, which corresponds to the animals' active period. The decrease in PER2 accompanied by increased c-Fos at ZT0 in the TMT0 group may reflect a change in timing of the physiological properties of these neurons in order to be prepared to respond to an expected fear-inducing stimulus in a time when the animal would normally be less active at the start of it's rest period. In comparison, PER2 in the TMT12 group was not affected at ZT0 but was instead increased at ZT 6 and ZT 12 in comparison to control animals. In addition, c-Fos expression was not altered at ZT 6 or ZT 12. This may reflect a change in timing in these neurons, and/or a small effect of the control procedure (cage movement and KimWipe without TMT) at ZT 0. Despite the change in PER2 expression during what would correspond to a large portion of the animals' inactive period, no change in cell activity indicated by c-Fos was detected in this group at ZT 6 or ZT 12. Animals in the TMT12 group may be less affected by TMT exposure at this timepoint possibly because they are beginning their active period at this time and are already alert and prepared to respond to fear stimuli.

PER2 expression in the CE was similar to that for the BLA in the experimental TMT groups. The expression of c-Fos however differed from the BLA in that there was no increase in c-Fos at ZT 0 for the TMT12 group, but there was a significant increase at ZT 12 for this group, indicating that unlike the BLA, the CE was preferentially activated at the time of TMT exposure. This observed increase in c-Fos expression specifically at the ZT of prior TMT exposure indicates that changes in expression are likely occurring in response to entrainment to the fear-inducing odor rather that to disturbances every 12 hours. Previous work in rats has shown that both innate and conditioned fear response increase c-Fos expression in the amygdala, including the BLA and the CE [76], [77]. In addition, other work has shown that fox urine also induces c-Fos expression in the amygdala, although induction was stronger in the BLA than the CE [57]. This study however measured immediate response to fox urine, rather than a circadian response of repeated exposure to a fear-inducing stimulus. In addition, animals in that study were exposed to fox urine at either ZT 6 or ZT 18, two timepoints that were not used for TMT exposure in our study. As indicated by our data, ZT may have a large effect on the amount of c-Fos induction in response to a fear-inducing odor.

Taken together, our results indicate that the amygdala, when compared to the PirCx, distinguishes TMT as a fear-inducing stimulus rather than a general odor, as indicated in the PirCx by the increase of c-Fos at both ZT 0 and ZT 12 in the TMT0 and TMT12 groups, in essence inducing a 12 hr rhythm in c-Fos in response to odor stimulation. Since c-Fos responses were similar for both groups (TMT0 and TMT12), we cannot exclude the possibility that the procedure alone and presence of the investigator caused the changes in c-Fos expression. In comparison, the CE, which acts as the output area of the amygdala to the hypothalamic-pituitary-adrenal axis (HPA), showed increased c-Fos only at ZT 0 for the TMT0 group and only at ZT 12 for the TMT12 group. This result corresponds with previously published work showing that both fox urine and mineral oil equally induce c-Fos in the PirCx, but fox urine induces a greater amount of c-Fos in the amygdala [57], indicating that, as in our study, the amygdala differentiates between a fearful and non fearful odor whereas the PirCx does not.

The observed effects in the BLA and CE, together with the lack of changes in PER2 and c-Fos in the PirCx, indicate that time-stamp fear memory for the TMT odor is occurring primarily in the amygdala, and more strongly at ZT 0 than ZT 12. This interpretation is supported by studies showing that fear memory encoding, reconsolidation, and retrieval occurs in the amygdala, [62], [63], [64], [65], [66], and this area is also essential for odor aversion memory consolidation [75]. This stronger effect of repeated TMT exposure at ZT 0 in comparison to ZT 12 indicates that fear memory formation may be stronger at this timepoint. Behavioral work has shown that mice acquire fear memory faster and retain it longer when trained during the day at ZT 3 than during the night at ZT 15 [29]. Our observed changes in PER2 and c-Fos in the BLA and CE at ZT 0 may underlie these behavioral observations that fear memory formation in mice is stronger during their inactive period.

Studies indicate that rhythmic expression of PER2 in the CE but not the BLA or the Hipp, is dependant upon rhythmic glucocorticoid expression [44], [78]. It is possible that the rhythm in glucocorticoid expression may in part explain why PER2 and c-Fos effects after repeated TMT exposure were stronger at ZT 0. Plasma corticosterone level peaks right before activity onset and remains high during the active period of rodents and is low at the beginning of the inactive period, corresponding to ZT 0 [79]. Glucocorticoids have been shown to affect the expression of circadian genes in peripheral tissues and fibroblasts [80]. In addition, glucocorticoid expression can enhance memory formation in part processed through the amygdala [81], [82], [83], [84], [85]. It is possible that during the early subjective day, a low level of plasma corticosterone in normal conditions allows for a larger effect of an increase of corticosterone in response to a fear-inducing stimulus, and in turn results in enhanced memory formation and possibly changes in circadian gene expression.

In summary, repeated exposure to a fear-inducing odor alters rhythmic expression of PER2 and induces c-Fos expression in selective brain areas involved in processing this information. Changes in PER2 and c-Fos expression were seen 24 hours after the last exposure to TMT, indicating that they were not acute responses to the stimulus. This is consistent with entrainment of endogenous rhythms in separate oscillators in these regions. The entrainment of these separate oscillators may be part of the mechanism behind the formation of time-stamp memories. Animals exposed to the fear-inducing odor during the early day were affected much more than animals exposed during the early night, indicating that changes in PER2 and c-Fos expression in selective brain regions may underlie the reported improved acquisition and retention of fear memory during the subjective day in comparison to the subjective night in rodents [29].

## References

[1] Moore RY (1997). Circadian rhythms: basic neurobiology and clinical applications.. Annu Rev Med.

[2] Hastings MH, Herzog ED (2004). Clock genes, oscillators, and cellular networks in the suprachiasmatic nuclei.. J Biol Rhythms.

[3] Reppert SM, Weaver DR (2002). Coordination of circadian timing in mammals.. Nature.

[4] Ko CH, Takahashi JS (2006). Molecular components of the mammalian circadian clock.. Hum Mol Genet 15 Spec No.

[5] Lamia KA, Storch KF, Weitz CJ (2008). Physiological significance of a peripheral tissue circadian clock.. Proc Natl Acad Sci U S A.

[6] Storch KF, Lipan O, Leykin I, Viswanathan N, Davis FC (2002). Extensive and divergent circadian gene expression in liver and heart.. Nature.

[7] Schibler U, Ripperger J, Brown SA (2003). Peripheral circadian oscillators in mammals: time and food.. J Biol Rhythms.

[8] Li Z, Ruan L, Lin S, Gittes GK (2007). Clock controls timing of mouse pancreatic differentiation through regulation of Wnt- and Notch-based and cell division components.. Biochem Biophys Res Commun.

[9] Gibbs JE, Beesley S, Plumb J, Singh D, Farrow S (2009). Circadian timing in the lung; a specific role for bronchiolar epithelial cells.. Endocrinology.

[10] Bando H, Nishio T, van der Horst GT, Masubuchi S, Hisa Y (2007). Vagal regulation of respiratory clocks in mice.. J Neurosci.

[11] Brown SA, Fleury-Olela F, Nagoshi E, Hauser C, Juge C (2005). The period length of fibroblast circadian gene expression varies widely among human individuals.. PLoS Biol.

[12] Geyfman M, Andersen B (2009). How the skin can tell time.. J Invest Dermatol.

[13] Frigato E, Lunghi L, Ferretti ME, Biondi C, Bertolucci C (2009). Evidence for circadian rhythms in human trophoblast cell line that persist in hypoxia.. Biochem Biophys Res Commun.

[14] Sothern RB, Cornelissen G, Yamamoto T, Takumi T, Halberg F (2009). Time microscopy of circadian expression of cardiac clock gene mRNA transcription: chronodiagnostic and chrono-therapeutic implications.. Clin Ter.

[15] Leibetseder V, Humpeler S, Svoboda M, Schmid D, Thalhammer T (2009). Clock genes display rhythmic expression in human hearts.. Chronobiol Int.

[16] Vollmers C, Gill S, DiTacchio L, Pulivarthy SR, Le HD (2009). Time of feeding and the intrinsic circadian clock drive rhythms in hepatic gene expression.. Proc Natl Acad Sci U S A.

[17] Sherman H, Frumin I, Gutman R, Chapnik N, Lorentz A Long-term restricted feeding alters circadian expression and reduces the level of inflammatory and disease markers.. J Cell Mol Med.

[18] Doi R, Oishi K. Ishida N CLOCK regulates circadian rhythms of hepatic glycogen synthesis through transcriptional activation of Gys2.. J Biol Chem.

[19] Storch KF, Paz C, Signorovitch J, Raviola E, Pawlyk B (2007). Intrinsic circadian clock of the mammalian retina: importance for retinal processing of visual information.. Cell.

[20] Beling I (1929). Uber das Zeitgedachtnis der Bienen.. Zeitschrift fur Vergleichendre Physiologie.

[21] Fuller PM, Lu J, Saper CB (2008). Differential rescue of light- and food-entrainable circadian rhythms.. Science.

[22] Carneiro BT, Araujo JF (2009). The food-entrainable oscillator: a network of interconnected brain structures entrained by humoral signals?. Chronobiol Int.

[23] Verwey M, Amir S (2009). Food-entrainable circadian oscillators in the brain.. Eur J Neurosci.

[24] Mistlberger RE (2009). Food-anticipatory circadian rhythms: concepts and methods.. Eur J Neurosci.

[25] Verwey M, Khoja Z, Stewart J, Amir S (2007). Differential regulation of the expression of Period2 protein in the limbic forebrain and dorsomedial hypothalamus by daily limited access to highly palatable food in food-deprived and free-fed rats.. Neuroscience.

[26] O'Brien J, Sutherland RJ (2007). Evidence for episodic memory in a pavlovian conditioning procedure in rats.. Hippocampus.

[27] Cain SW, McDonald RJ, Ralph MR (2008). Time stamp in conditioned place avoidance can be set to different circadian phases.. Neurobiol Learn Mem.

[28] Cain SW, Ralph MR (2009). Circadian modulation of conditioned place avoidance in hamsters does not require the suprachiasmatic nucleus.. Neurobiol Learn Mem.

[29] Chaudhury D, Colwell CS (2002). Circadian modulation of learning and memory in fear-conditioned mice.. Behav Brain Res.

[30] Cain SW, Chou T, Ralph MR (2004). Circadian modulation of performance on an aversion-based place learning task in hamsters.. Behav Brain Res.

[31] Loh DH, Navarro J, Hagopian A, Wang LM, Deboer T Rapid changes in the light/dark cycle disrupt memory of conditioned fear in mice.. PLoS One.

[32] Gerstner JR, Lyons LC, Wright KP, Loh DH, Rawashdeh O (2009). Cycling behavior and memory formation.. J Neurosci.

[33] Lamont EW, Robinson B, Stewart J, Amir S (2005). The central and basolateral nuclei of the amygdala exhibit opposite diurnal rhythms of expression of the clock protein Period2.. Proc Natl Acad Sci U S A.

[34] Wang LM, Dragich JM, Kudo T, Odom IH, Welsh DK (2009). Expression of the circadian clock gene Period2 in the hippocampus: possible implications for synaptic plasticity and learned behaviour.. ASN Neuro.

[35] Granados-Fuentes D, Tseng A, Herzog ED (2006). A circadian clock in the olfactory bulb controls olfactory responsivity.. J Neurosci.

[36] Gottfried JA Central mechanisms of odour object perception.. Nat Rev Neurosci.

[37] LeDoux JE (2000). Emotion circuits in the brain.. Annu Rev Neurosci.

[38] Seidenbecher T, Laxmi TR, Stork O, Pape HC (2003). Amygdalar and hippocampal theta rhythm synchronization during fear memory retrieval.. Science.

[39] Endres T, Fendt M (2009). Aversion- vs fear-inducing properties of 2,4,5-trimethyl-3-thiazoline, a component of fox odor, in comparison with those of butyric acid.. J Exp Biol.

[40] Pagani JH, Rosen JB (2009). The medial hypothalamic defensive circuit and 2,5-dihydro-2,4,5-trimethylthiazoline (TMT) induced fear: comparison of electrolytic and neurotoxic lesions.. Brain Res.

[41] Buron G, Hacquemand R, Pourie G, Lucarz A, Jacquot L (2007). Comparative behavioral effects between synthetic 2,4,5-trimethylthiazoline (TMT) and the odor of natural fox (Vulpes vulpes) feces in mice.. Behav Neurosci.

[42] King JA, De Oliveira WL, Patel N (2005). Deficits in testosterone facilitate enhanced fear response.. Psychoneuroendocrinology.

[43] Wallace KJ, Rosen JB (2000). Predator odor as an unconditioned fear stimulus in rats: elicitation of freezing by trimethylthiazoline, a component of fox feces.. Behav Neurosci.

[44] Segall LA, Milet A, Tronche F, Amir S (2009). Brain glucocorticoid receptors are necessary for the rhythmic expression of the clock protein, PERIOD2, in the central extended amygdala in mice.. Neurosci Lett.

[45] Sotres-Bayon F, Cain CK, LeDoux JE (2006). Brain mechanisms of fear extinction: historical perspectives on the contribution of prefrontal cortex.. Biol Psychiatry.

[46] Phelps EA, LeDoux JE (2005). Contributions of the amygdala to emotion processing: from animal models to human behavior.. Neuron.

[47] Lamont EW, Robinson B, Stewart J, Amir S (2005). The central and basolateral nuclei of the amygdala exhibit opposite diurnal rhythms of expression of the clock protein Period2.. Proceedings of the National Academy of Sciences of the United States of America.

[48] Amir S, Stewart J (2009). Motivational Modulation of Rhythms of the Expression of the Clock Protein PER2 in the Limbic Forebrain.. Biological psychiatry.

[49] Kudo T, Loh DH, Kuljis D, Constance C, Colwell CS (2011). Fast delayed rectifier potassium current: critical for input and output of the circadian system.. The Journal of neuroscience : the official journal of the Society for Neuroscience.

[50] Hughes AT, Fahey B, Cutler DJ, Coogan AN, Piggins HD (2004). Aberrant gating of photic input to the suprachiasmatic circadian pacemaker of mice lacking the VPAC2 receptor.. J Neurosci.

[51] Miller JP, McAuley JD, Pang KC (2005). Spontaneous fos expression in the suprachiasmatic nucleus of young and old mice.. Neurobiology of aging.

[52] Sumova A, Travnickova Z, Mikkelsen JD, Illnerova H (1998). Spontaneous rhythm in c-Fos immunoreactivity in the dorsomedial part of the rat suprachiasmatic nucleus.. Brain research.

[53] Li W, Howard JD, Gottfried JA. Disruption of odour quality coding in piriform cortex mediates olfactory deficits in Alzheimer's disease.. Brain.

[54] Howard JD, Plailly J, Grueschow M, Haynes JD, Gottfried JA (2009). Odor quality coding and categorization in human posterior piriform cortex.. Nat Neurosci.

[55] Suzuki N, Bekkers JM. Distinctive Classes of GABAergic Interneurons Provide Layer-Specific Phasic Inhibition in the Anterior Piriform Cortex.. Cereb Cortex.

[56] Stettler DD, Axel R (2009). Representations of odor in the piriform cortex.. Neuron.

[57] Funk D, Amir S (2000). Circadian modulation of fos responses to odor of the red fox, a rodent predator, in the rat olfactory system.. Brain Res.

[58] Squire LR (1992). Memory and the hippocampus: a synthesis from findings with rats, monkeys, and humans.. Psychol Rev.

[59] Fortin NJ, Wright SP, Eichenbaum H (2004). Recollection-like memory retrieval in rats is dependent on the hippocampus.. Nature.

[60] Eichenbaum H (2004). Hippocampus: cognitive processes and neural representations that underlie declarative memory.. Neuron.

[61] Chaudhury D, Wang LM, Colwell CS (2005). Circadian regulation of hippocampal long-term potentiation.. J Biol Rhythms.

[62] Doyere V, Debiec J, Monfils MH, Schafe GE, LeDoux JE (2007). Synapse-specific reconsolidation of distinct fear memories in the lateral amygdala.. Nat Neurosci.

[63] Reijmers LG, Perkins BL, Matsuo N, Mayford M (2007). Localization of a stable neural correlate of associative memory.. Science.

[64] Gale GD, Anagnostaras SG, Godsil BP, Mitchell S, Nozawa T (2004). Role of the basolateral amygdala in the storage of fear memories across the adult lifetime of rats.. J Neurosci.

[65] Debiec J, Doyere V, Nader K, Ledoux JE (2006). Directly reactivated, but not indirectly reactivated, memories undergo reconsolidation in the amygdala.. Proc Natl Acad Sci U S A.

[66] Debiec J, Diaz-Mataix L, Bush DE, Doyere V, Ledoux JE. The amygdala encodes specific sensory features of an aversive reinforcer.. Nat Neurosci.

[67] Debiec J, Ledoux JE (2004). Disruption of reconsolidation but not consolidation of auditory fear conditioning by noradrenergic blockade in the amygdala.. Neuroscience.

[68] Kajiwara R, Tominaga T, Takashima I (2007). Olfactory information converges in the amygdaloid cortex via the piriform and entorhinal cortices: observations in the guinea pig isolated whole-brain preparation.. Eur J Neurosci.

[69] Luskin MB, Price JL (1983). The topographic organization of associational fibers of the olfactory system in the rat, including centrifugal fibers to the olfactory bulb.. J Comp Neurol.

[70] Ottersen OP (1982). Connections of the amygdala of the rat. IV: Corticoamygdaloid and intraamygdaloid connections as studied with axonal transport of horseradish peroxidase.. J Comp Neurol.

[71] Pikkarainen M, Ronkko S, Savander V, Insausti R, Pitkanen A (1999). Projections from the lateral, basal, and accessory basal nuclei of the amygdala to the hippocampal formation in rat.. J Comp Neurol.

[72] Pitkanen A, Pikkarainen M, Nurminen N, Ylinen A (2000). Reciprocal connections between the amygdala and the hippocampal formation, perirhinal cortex, and postrhinal cortex in rat. A review.. Ann N Y Acad Sci.

[73] Pitkanen A, Kelly JL, Amaral DG (2002). Projections from the lateral, basal, and accessory basal nuclei of the amygdala to the entorhinal cortex in the macaque monkey.. Hippocampus.

[74] Benes FM, Berretta S (2000). Amygdalo-entorhinal inputs to the hippocampal formation in relation to schizophrenia.. Ann N Y Acad Sci.

[75] Desgranges B, Levy F, Ferreira G (2008). Anisomycin infusion in amygdala impairs consolidation of odor aversion memory.. Brain Res.

[76] Holahan MR, White NM (2004). Amygdala c-Fos induction corresponds to unconditioned and conditioned aversive stimuli but not to freezing.. Behav Brain Res.

[77] Campeau S, Hayward MD, Hope BT, Rosen JB, Nestler EJ (1991). Induction of the c-fos proto-oncogene in rat amygdala during unconditioned and conditioned fear.. Brain Res.

[78] Segall LA, Perrin JS, Walker CD, Stewart J, Amir S (2006). Glucocorticoid rhythms control the rhythm of expression of the clock protein, Period2, in oval nucleus of the bed nucleus of the stria terminalis and central nucleus of the amygdala in rats.. Neuroscience.

[79] Oster H, Damerow S, Kiessling S, Jakubcakova V, Abraham D (2006). The circadian rhythm of glucocorticoids is regulated by a gating mechanism residing in the adrenal cortical clock.. Cell Metab.

[80] Balsalobre A, Brown SA, Marcacci L, Tronche F, Kellendonk C (2000). Resetting of circadian time in peripheral tissues by glucocorticoid signaling.. Science.

[81] Roozendaal B, McGaugh JL (1997). Basolateral amygdala lesions block the memory-enhancing effect of glucocorticoid administration in the dorsal hippocampus of rats.. Eur J Neurosci.

[82] McGaugh JL, Cahill L, Roozendaal B (1996). Involvement of the amygdala in memory storage: interaction with other brain systems.. Proc Natl Acad Sci U S A.

[83] Thompson BL, Erickson K, Schulkin J, Rosen JB (2004). Corticosterone facilitates retention of contextually conditioned fear and increases CRH mRNA expression in the amygdala.. Behav Brain Res.

[84] Nathan SV, Griffith QK, McReynolds JR, Hahn EL, Roozendaal B (2004). Basolateral amygdala interacts with other brain regions in regulating glucocorticoid effects on different memory functions.. Ann N Y Acad Sci.

[85] Roozendaal B, McEwen BS, Chattarji S (2009). Stress, memory and the amygdala.. Nat Rev Neurosci.

